# The LacI–Family Transcription Factor, RbsR, Is a Pleiotropic Regulator of Motility, Virulence, Siderophore and Antibiotic Production, Gas Vesicle Morphogenesis and Flotation in *Serratia*

**DOI:** 10.3389/fmicb.2017.01678

**Published:** 2017-09-11

**Authors:** Chin M. Lee, Rita E. Monson, Rachel M. Adams, George P. C. Salmond

**Affiliations:** Department of Biochemistry, University of Cambridge Cambridge, United Kingdom

**Keywords:** gas vesicles, *Serratia*, virulence, ribose operon, motility, antibiotics, gene regulation

## Abstract

Gas vesicles (GVs) are proteinaceous, gas-filled organelles used by some bacteria to enable upward movement into favorable air/liquid interfaces in aquatic environments. *Serratia* sp. ATCC39006 (S39006) was the first enterobacterium discovered to produce GVs naturally. The regulation of GV assembly in this host is complex and part of a wider regulatory network affecting various phenotypes, including antibiotic biosynthesis. To identify new regulators of GVs, a comprehensive mutant library containing 71,000 insertion mutants was generated by random transposon mutagenesis and 311 putative GV-defective mutants identified. Three of these mutants were found to have a transposon inserted in a LacI family transcription regulator gene (*rbsR*) of the putative ribose operon. Each of these *rbsR* mutants was GV-defective; no GVs were visible by phase contrast microscopy (PCM) or transmission electron microscopy (TEM). GV deficiency was caused by the reduction of *gvpA1* and *gvrA* transcription (the first genes of the two contiguous operons in the GV gene locus). Our results also showed that a mutation in *rbsR* was highly pleiotropic; the production of two secondary metabolites (carbapenem and prodigiosin antibiotics) was abolished. Interestingly, the intrinsic resistance to the carbapenem antibiotic was not affected by the *rbsR* mutation. In addition, the production of a siderophore, cellulase and plant virulence was reduced in the mutant, whereas it exhibited increased swimming and swarming motility. The RbsR protein was predicted to bind to regions upstream of at least 18 genes in S39006 including *rbsD* (the first gene of the ribose operon) and *gvrA*. Electrophoretic mobility shift assays (EMSA) confirmed that RbsR bound to DNA sequences upstream of *rbsD*, but not *gvrA*. The results of this study indicate that RbsR is a global regulator that affects the modulation of GV biogenesis, but also with complex pleiotropic physiological impacts in S39006.

## Introduction

The capacity to move is an important ecological adaptation in bacteria. Bacteria are exposed to constantly changing environments and so mobility provides potential advantages for survival. In response to environmental cues, several mobility methods, such as swimming, swarming, gliding, twitching, and floating, are used by prokaryotes to propel themselves into favorable niches (Jarrell and McBride, 2008). Flotation, using gas vesicles (GVs), was first discovered in cyanobacteria over a century ago (Klebahn, 1895) but research on the molecular biology of GV regulation remains comparatively underexplored.

GVs are proteinaceous gas-filled intracellular organelles that facilitate buoyancy. They are synthesized by aquatic Eubacteria and Archaea (Pfeifer, 2012). GVs are spindle- or cylinder-shaped structures comprised of a thin proteinaceous wall (Pfeifer, 2012). The wall of the GV is freely permeable to dissolved gases such as oxygen, carbon dioxide, nitrogen, and methane that are present in the environment (Walsby, 1982). GVs reduce overall cell density and thereby enable bacterial cells to float and colonize air-liquid interfaces for enhanced fitness. Recently, the discovery of GVs in the genetically-amenable enterobacterium, *Serratia* sp. ATCC39006 (S39006), was reported (Ramsay et al., 2011; Tashiro et al., 2016).

S39006 is a Gram-negative, motile, rod-shaped bacterium that, in addition to flotation, exhibits flagellum-mediated swarming and swimming motility (Williamson et al., 2008; Ramsay et al., 2011). This strain produces various secondary metabolite antibiotics, including a tripyrrole, red pigment, 2-methyl-3-pentyl-6-methoxyprodigiosin (prodigiosin; a prodiginine), and the β-lactam antibiotic, 1-carbapen-2-em-3-carboxylic acid (a carbapenem) (Coulthurst et al., 2005; Williamson et al., 2006). S39006 is pathogenic to potato, secreting extracellular plant cell wall degrading enzymes (PCWDEs), such as pectate lyase and cellulase (Fineran et al., 2007). This bacterium also kills the nematode worm, *Caenorhabditis elegans* (Coulthurst et al., 2004). Many of these characteristics are under the control of quorum sensing (QS) which is the bacterial cell-cell communication system that controls gene expression in response to population density (Thomson et al., 2000).

**Figure 1:**
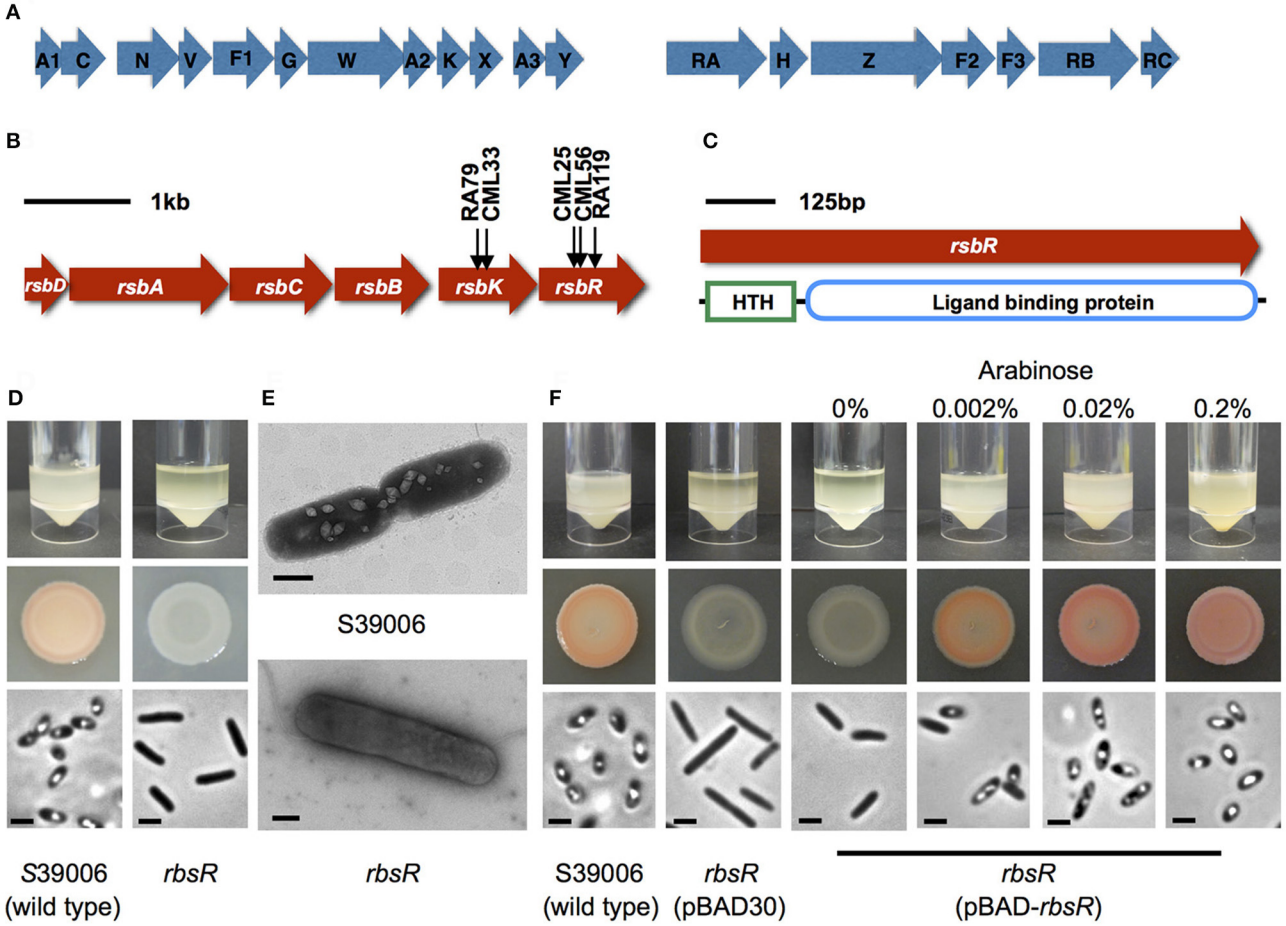
The *rbsR* mutation affects the GV phenotype in S39006. **(A)** The genetic organization of the gas vesicle genetic cluster in S39006. The first operon begins with *gvpA1* and the second with *gvrA*. Regulatory genes are indicated with an R. **(B)** The genetic organization of the ribose operon in S39006. The arrows indicate the positions of transposon insertions identified in this study. **(C)** Predicted protein domains in the *rbsR* amino acid sequences. **(D)** Comparison of the wild type and the *rbsR* mutant. Flotation assays of the wild type and the *rbsR* mutant 48 h after inoculation (top image). Bacterial culture spot test on plates with normalized cell number (middle image). PCM images from bacterial colonies on agar plates; the scale bar represents 1 μm (bottom image). **(E)** Representative TEM images showing the *rsbR* mutant with no GVs. The scale bar at the right bottom represents 500 nm. **(F)** The effect of ectopic expression of *rbsR* from pBAD-*rbsR* in the *rbsR* mutant at the indicated concentrations of arabinose. The top image indicates flotation assays of the wild type, the *rbsR* mutant carrying the empty plasmid or the *rbsR* mutant carrying pBAD-*rbsR* 48 h after inoculation. The middle image shows bacterial patches on plates with normalized cell number. The bottom image shows PCM images from bacterial colonies on an agar plates; the scale bar represents 1 μm.

The S39006 GV gene locus of 16.6 kb comprises 19 open reading frames (ORFs) organized into two contiguous operons (Ramsay et al., 2011) (Figure 1A). Three genes (*gvpA1, A2*, and *A3)* encode homologs of the GV major structural protein—GvpA in other proteobacteria. In addition, the GV gene cluster also encodes a homolog of GvpC which is a vesicle outer surface protein that plays a critical role in strengthening the GV. The GV gene locus also encodes minor structural proteins GvpF1-F3. The main regulatory proteins affecting GV biogenesis are encoded by *gvrA-C*. Other GV-associated proteins encoded by the cluster include GvpG, GvpH, GvpK, and GvpN. Finally, the locus also encodes proteins of unknown function—GvpV, GvpW, GvpX, GvpY, and GvpZ (Figure 1A). Recent evidence demonstrated that 11 genes (*gvpA1, gvpF1, gvpG, gvpA2, gvpK, gvpA3, gvpF2, gvpF3, gvrA, gvrB*, and *gvrC*) were essential for morphogenesis of GVs in S39006. In addition, certain GV proteins need to be maintained in correct stoichiometry ratios; GV production was significantly reduced when protein GvpF1, GvpF2, GvrA, GvrB, or GvrC was in excess. No GVs were produced when *gvpV* and *gvpA3* were overexpressed (Monson et al., 2016).

QS and oxygen availability are known cues controlling GV production. A *smaI* mutant that is unable to produce *N*-butanoyl-L-homoserine lactone (BHL, a QS molecule) fails to make GVs and the transcription of *gvpA1* is greatly increased in oxygen-limited batch culture, during stationary phase. RsmA, the global post-transcriptional regulator of secondary metabolism, also controls GV production (Ramsay et al., 2011). The regulation of GV biogenesis must be a highly coordinated process involving multiple environmental and physiological inputs but the extent of the GV regulatory network is not fully understood (Ramsay and Salmond, 2012). Consequently, in this study we decided to search for new inputs to the regulation of of GV morphogenesis.

To identify novel regulators of GVs in S39006, we employed random transposon mutagenesis to screen for mutants defective in GV production. From these experiments, three mutants were obtained with insertions in *rbsR* and two in *rbsK*, the gene immediately upstream of *rbsR*. Each *rbsR* mutant lost the ability to produce GVs and was also defective for production of the two bioactive secondary metabolites (the carbapenem and prodigiosin antibiotics), but *rbsR* mutants also exhibited increased swimming and swarming motility. The extensive pleiotropy of the mutant was further demonstrated by impacts on cellulase production, siderophore elaboration, and virulence.

## Materials and methods

### Bacterial strains, plasmids, phage, and culture conditions

Bacterial strains, plasmids, and phage used in this study are listed in Table 1. S39006 strains were grown at 30°C and *Escherichia coli* strains were grown at 37°C in sealed plastic universals containing Lysogeny Broth (LB; 5 g l^−1^ yeast extract, 10 g l^−1^ tryptone, and 5 g l^−1^ NaCl) or on solid LB agar plate (LBA) supplemented with 1.5% (w/v) agar. Bacterial growth was measured in a Unicam Heλios spectrophotometer and expressed as OD_600_. Where required, antibiotics were added at the following final concentrations: kanamycin (Kn), 50 μg ml^−1^, ampicillin (Ap), 100 μg ml^−1^, and chloramphenicol (Cm), 25 μg ml^−1^.

**Table 1:** Bacterial strains, plasmids. and phage used in the present study.

**Strain/phage/plasmid**	**Genotype/phenotype**	**References**
***E. coli***		
DH5α	F^−^Φ80*lac*ZΔM15 Δ(*lac*ZYA^−^*arg*F) U169 *rec*A1 *end*A1 *hsd*R17 (rK^−^, mK^+^) *pho*A *sup*E44 λ^−^*thi*-1	Life technology
β2163	*gyr*A96 *rel*A1 F^−^ RP4-2-Tc::Mu *dapA*::(*erm-pir*), Km^R^Em^R^	Demarre et al., 2005
ESS	β-lactam super sensitive strain	Bainton et al., 1992
***Serratia*** **sp**.		
S39006 (WT)	Lac^−^ strain derived from ATCC 39006	Thomson et al., 2000
NWA19	*lacA, ΔpigC*	Ramsay et al., 2011
GPA1	*gvpA1*:: *uidA*, Cm^R^	Ramsay et al., 2011
GRA	*gvrA*:: *uidA*, Cm^R^	Ramsay et al., 2011
LIS	*smaI::*mini-Tn*5*Sm/Sp, Sp^R^	Thomson et al., 2000
SP19	*smaI::*mini-Tn*5*Sm/Sp, *pigX*::Tn-DS1028, *pigZ*:: mini-Tn5*lacZ1*, Sp^R^, Cm^R^, Km^R^	Poulter et al., 2010
MCA54	*carA::*mini-Tn5*lacZ1*, Km^R^	Thomson et al., 2000
CML25	*rsbR::*mini-Tn5*lacZ1*, Km^R^	This study
CML26	*rsbR::*mini-Tn5*lacZ1*, Km^R^	This study
RA119	*rsbR::*mini-Tn5*lacZ1*, Km^R^	This study
CML33	*rsbK::*mini-Tn5*lacZ1*, Km^R^	This study
RA79	*rsbK::*mini-Tn5*lacZ1*, Km^R^	This study
***Pectobacterium carotovorum***		
ATTn10	ATCC 39048 carrying a Tn*10* insertion	McGowan et al., 1996
SM10	ATTn*10* deleted for Δ*carRABCDEFGH*	McGowan et al., 1997
**PHAGE**		
φOT8	*Serratia* generalized transducing phage	Evans et al., 2010
**PLASMID**		
pKRCPN1	Derivative of pDS1028*uidA* with the *uidA* and *cat* genes replaced with *lacZ* and *aph* genes. Km^R^, Tc^R^	Monson et al., 2015
pQE80-*oriT*	Expression vector for native or N-terminal hexa-histidine proteins containing the RK2 origin of transfer cloned as an *Nde*I fragment, Ap^R^	Ramsay et al., 2011
pQE-*rbsR*	pQE80-*oriT* carrying the hexa-histidine tagged *rbsR*	This study
pBAD30	Expression vector with *araBAD* promoter, Ap^R^	Guzman et al., 1995
pBAD-*rbsR*	pBAD30 carrying the wild type S39006 *rbsR*	This study
pBAD33	Expression vector with *araBAD* promoter, Cm^R^	Guzman et al., 1995
pBAD-*rbsK*	pBAD33 carrying the wild type S39006 *rbsK*	This study

To study growth under aeration conditions, 25 ml LB in 250 ml Erlenmeyer flasks were inoculated with an overnight culture of the test strain to an OD_600_ of 0.05. The culture was incubated at 30°C with shaking at 215 rpm. Sampling was carried out every 2 h. Similar procedures were used to study growth under microaerophilic conditions except that 25 ml mineral oil was overlaid on 25 ml LB and the flask was shaken at 80 rpm. For flotation assays, experiments were carried out as described in Tashiro et al. (2016). For bacterial spot tests, cultures were normalized to OD_600_ of 1 and 10 μl was spotted on LBA. Transduction was carried out as describe by Evans et al. (2010) using φOT8. Transductants were selected on LBA supplemented with Kn.

### Transposon mutagenesis

Transposon mutagenesis of S39006 was carried out as described previously (Monson et al., 2015). Briefly, the plasmid pKRCPN1 was delivered to the recipient by conjugation with *E. coli* β2163. A mating patch containing a ratio of 3:1 S39006 or LacA Δ*pigC* and *E. coli* β2163 was prepared and incubated overnight on LBA supplemented with 300 μM diaminopimelic acid (DAPA). Samples from the patch were serially diluted and plated on LBA plates containing Kn. The plates were incubated at 30°C for 48 h. Transconjugants were screened visually for their colony appearance. S39006 colonies are normally opaque but become translucent when they lose their ability to make GVs (Ramsay et al., 2011). The insertion site of the transposon in translucent mutants was then identified by random priming PCR (RP-PCR) analysis (Jacobs et al., 2003) followed by DNA sequencing across the transposon junction using oligos MAMV1-KRCN1 and MAMV2-KRCN1 (Table 2).

**Table 2:** Sequences of oligonucleotides used in this study.

**Name**	**5′–3′ sequence**	**Usage**	**References**
PF106	GACCACACGTCGACTAGTGCNNNNNNNNNNAGAG	Random priming PCR	Fineran et al., 2007
PF107	GACCACACGTCGACTAGTGCNNNNNNNNNNACGCC	Random priming PCR	Fineran et al., 2007
PF108	GACCACACGTCGACTAGTGCNNNNNNNNNNGATAC	Random priming PCR	Fineran et al., 2007
PF109	GACCACACGTCGACTAGTGC	Random priming PCR	Fineran et al., 2007
MAMV1-KRCPN1	GGAATTGATCCGGTGGATG	Transposon specific oligo for TnDS1028	Matilla et al., 2012
MAMV2-KRCPN1	GCATAAAGCTTGCTCAATCAATCAC	Transposon specific oligo for TnDS1028	Matilla et al., 2012
oCML24	TTATCAAAGCTTGCTTATAGCGGAGTATATGAGG	Cloning of *rbsR* into pQE80-*oriT* or pBAD30 (restriction site—*Hin*dIII)	This study
oCML36	CTCTCAGGTACCATGAAAGATGTTGCCCGTC	Cloning of *rbsR* into pQE80-*oriT* (restriction site—*Kpn*I)	This study
oCML37	CTCTCAGGTACCGCACAGGGGTGATCTTTG	Cloning of *rbsR* into pBAD30 (restriction site—*Kpn*I)	This study
oMC100	CATCATCATCATCATCATCATCATCATCATCAT	Non-specific oligo short—EMSA	This study
oMC101	CATCATCATCATCATCATCATCATCATCATCATTCCAGACCAGGGCAC	Non-specific oligo long—EMSA	This study
oREM726	CGCGGGTACCAGTGGCACACGATTAACTTTGGG	Cloning of *rbsK* into pBAD33 (restriction site *KpnI*)	This study
oREM727	CGCGAAGCTTTCACCCCTGTGCTTGCAAGAAA	Cloning of *rbsK* into pBAD33 (restriction site *Hin*dIII)	This study

### Measurement of β-glucuronidase and β-galactosidase activity

β-glucuronidase (β-glu) activity was measured as described by Ramsay et al. (2011). A 100 μl sample of culture was taken at each time point and frozen at −80°C. The sample was then thawed at room temperature. Phosphate-buffered saline (100 μl) containing 400 μg ml^−1^ lysozyme, 250 μg ml^−1^ 4′-methylumbelliferyl-β-D-glucuronide (MUG) was added to 10 μl of sample. The fluorescence emitted by the samples was then immediately monitored (excitation 360 nm, emission 450 nm, cut-off 435 nm, eight reads per well, measured every 30 s for 30 min at 37°C) using a Gemini XPS plate reader and expressed as RFU OD_600_^−1^. β-galactosidase activity was determined using similar procedure except that MUG was replaced by 4′-methylumbelliferyl-β-D-galactoside.

### Phenotypic assays

Phenotypic assays for siderophore, cellulase, pectate lyase, BHL, prodigiosin, and carbapenem production, plus swarming and swimming assays, were performed as described previously (Schwyn and Neilands, 1987; Slater et al., 2003; Williamson et al., 2008; Poulter et al., 2010). In all phenotypic plate assays, overnight cultures of the test strains were adjusted to an OD_600_ of 1.0, 10 μl (or 5 μl for swimming and swarming assays) spotted on appropriate agar plates and incubated at 30°C for 48 h. Indicator plates for BHL detection were prepared by adding 100 μl of *Serratia* biosensor strain SP19 with 3 ml of molten 0.75% agar. Detection of BHL was indicated by the production of a red halo by the biosensor strain around the test colonies (Poulter et al., 2010). Indicator plates for carbapenem production were made using 0.75% top agar lawns seeded with *E. coli* strain ESS. The production of antibiotic was indicated by the formation of inhibition zones around the test colonies (Slater et al., 2003). Phenotypic assay plates for cellulase production were developed by flooding with 0.2% (w/v) of congo red for 20 min, bleached with 1 M NaCl and finally stained with 1 M HCl for 5 min. For assessing pectate lyase activity, the agar plate was flooded with 7.5% (w/v) copper acetate for 1–2 h. Enzyme activity was indicated by halos formed around the test strain. Swimming and swarming plates were assessed visually. Swarming behavior varies between plates and therefore images of plates shown are representative of those observed. Comparisons of swarming behavior were always between strains swarming on the same plate to avoid plate-to-plate variation.

### Phase contrast microscopy and transmission electron microscopy

Phase contrast microscopy (PCM) images were obtained from wet mounts of bacterial samples from a colony or liquid culture, using an Olympus BX-51 with a 100X oil-immersion lens. For transmission electron microscopy (TEM), a carbon-coated glow discharge grid was treated with 0.01% poly-L-lysine (2 min) and the bacterial sample was attached to the grid for 10 min. The grids were washed twice with dH_2_O and stained with 2% phosphotungstic acid (pH 7.0) for 5 min. The grids were viewed using a FEI Tecnai G2 TEM in the Cambridge University Advanced Imaging Facility.

### Construction of plasmids

To construct pBAD-*rbsR* for complementation assays, the *rbsR* gene was first amplified using oligonucleotide pair, oCML24 and oCML37. Oligonucleotides used for PCR amplification were purchased from Sigma Aldrich and are listed in Table 2. The PCR product and plasmid pBAD30 were digested with *Kpn*I (NEB) and *Hin*dIII (NEB) at 37°C for 2 h. The digested PCR product was subsequently ligated into compatibly digested pBAD30 using T4 DNA ligase (NEB) according to the manufacturer's instructions. Plasmid pQE80-*rbsR* was constructed using oligonucleotides oCML24 and oCML36 to amplify the *rbsR* open reading frame (ORF). The amplified fragment was digested with *Kpn*I (NEB) and *Hin*dIII (NEB) and ligated with compatibility digested pQE80-*oriT*. For the construction of pBAD-*rbsK*, oligonucleotides oREM726 and oREM727 were used to amplify the *rbsK* ORF and the subsequent PCR product was digested with *Kpn*I and *Hin*dIII. This was ligated with compatibly digested pBAD33 to form pBAD-*rbsK*. All plasmids were subjected to sequencing (GATC Biotech) to confirm the sequence was correct.

### Protein expression and purification

The pQE80-*rbsR* plasmid was transferred into S39006 by conjugation using *E. coli* β2163 and RbsR production was induced by addition of 1 mM IPTG to the bacterial culture. The RbsR protein was purified using Ni-NTA agarose according to the manufacturer's instructions (Qiagen, Germany). The identity and nature of the purified protein was checked by SDS-PAGE and Western blot analysis. Protein concentration was measured by DC protein assay (Biorad) according to the manufacturer's instructions.

### Electrophoretic mobility shift assay (EMSA)

DNA probes for EMSA analyses were prepared according to the LUEGO method. The universal “third oligonucleotides” (LUEGO), which was fluorescently labeled at 5′ and 3′ ends, was used to generate many different probes (Jullien and Herman, 2011). To perform EMSA, oligonucleotide mixtures (5:5:1 LUEGO:short:long) were prepared and annealed in a thermocycler by the following profile: 2 mins at 95°C, cooled down to 70°C at 5°C s^−1^ followed by slow cooling at 0.02°C min^−1^ to 18°C. The mixture was then diluted to 5 nM and mixed with the indicated concentrations of the RbsR protein. This protein-probe mixture was added to the EMSA reaction buffer (10% glycerol, 10 mM Tris pH 8.0, 1 mM DTT, 10 mM KCl, 0.5 mM EDTA, and 20 μg m^−1^ BSA) to a total volume of 20 μl. The reaction was incubated on ice for 30 min then 10 μl of the sample were loaded onto a 7.5% native acrylamide 1X TGE **(**25 mM Tris pH 8.0, 192 mM Glycine, and 2 mM EDTA) gel and subjected to 100 V at 4°C. The gel was scanned using a Typhoon 9400 scanner with the following parameters: Acquisition mode: fluorescence, focal plane: +3 mm, emission filter: 670 BP30 Cy5, PMT: 600, laser: red (635 nm). Where indicated, a non-specific probe, made using oligonucleotides oMC100 and oMC101, was used. Together these oligos form a binding site consisting of 10 repeats of the nucleotides CAT (Table 2).

### Plant virulence assays

Potato rotting assays were performed as described by Fineran et al. (2007) to assess the virulence of the *rbsR* mutant in plants. Potatoes were inoculated with 1 × 10^6^ cfu of S39006 or the *rbsR* mutant. After 5 days incubation at 30°C, rotted tissue was weighed and compared. To assess colony counts, 0.1 gram of rotten potato tissue (or a normalized amount where the amount recovered was low) was serially diluted in LB. Serial dilutions were plated out onto LB and colony counts assessed. Statistical analyses were performed using a two-tailed *t*-test; differences were significant if the *P*-value < 0.05.

### Bioinformatics and phylogenetic analysis

The *rbsR* sequence was compared to those available in Genbank (Benson et al., 2013) using Basic Local Alignment Search Tool (BLAST) (Altschul et al., 1990). Conserved protein domains were analyzed using pfam (Bateman et al., 2004) and Conserved Domain Database (CCD) (Marchler-Bauer et al., 2014). The binding motifs of RbsR were predicted using the MEME suite (Bailey et al., 2009). The amino acid sequences of *rbsR* from *Serratia* and closely related strains were used to construct the phylogenetic tree using Molecular Evolutionary Genetic Analysis (MEGA) version 7.0 (Kumar et al., 2016). The evolution history was inferred by the Maximum Likelihood method based on the JTT matrix-based model (Jones et al., 1992). All positions containing gaps and missing data were eliminated. Bootstrap trials were replicated 1,000 times to estimate confidence values of the phylogenetic tree.

## Results

### Generation of transposon insertion mutant library

To identify new regulators of GV production, plasmid pKRCPN1 was first used to randomly mutagenize S39006. The plasmid contains a Tn*5* transposon derivative containing a promoterless *lacZ* gene and a kanamycin resistance cassette (mini-Tn*5*Kn*lacZ1*) (Monson et al., 2015). S39006 colonies are normally opaque because of light-refracting GVs in the bacterial cells. The bacterial colonies become translucent when GV production is inhibited and so presumptive GV-defective mutants could be identified based on their translucent colonial morphology on agar plates. However, visual screening for GV-deficient mutants in a strain making the red pigment prodigiosin proved problematic as definition of the translucent phenotype was sometimes ambiguous. To overcome this, S39006 LacA Δ*pigC*, a non-pigmented mutant carrying a mutation in *pigC*, was used for further transposon mutant screens.

A comprehensive mutant library containing 71,000 insertion mutants was generated by random transposon mutagenesis. From 67 independent conjugations, 311 putative GV-defective mutants were obtained. The transposon insertion sites in the mutants were determined by RP-PCR. Three of these GV-defective mutants (CML25, CML26, and RA119) had a transposon inserted in a putative LacI family transcription regulator gene (*or508* or *rbsR*) and two (CML33, RA79) had an insertion in the ribokinase gene (*rbsK*) of the ribose operon (Figure 1B) that is responsible for ribose transport and utilization in some bacteria. In addition to mutations in *rbsR* and *rbsK*, the screen also identified transposon insertions in two previously defined regulators of gas vesicle biosynthesis in S39006; namely *pigX* and *smaI* (Ramsay et al., 2011). Transposons insertions were also identified in *gvpA1* and *gvpN*—two genes that lie within the gas vesicle biosynthetic cluster and which we showed previously to be essential for robust gas vesicle formation (Tashiro et al., 2016).

### Sequence analysis and genomic context of *rbsR*

The ORF *or*508 (locus of transposon inserted in mutant strain CML26) of S39006 is predicted to encode the LacI family transcriptional regulator of the ribose operon. The amino acid sequence of *or*508 exhibited high similarity to several putative LacI family transcriptional regulators from other enterobacteria, including *Dickeya dadantii, D. dianthicola, D. solani, D. zeae, D. chrysanthemi*, and *Pectobacterium atrosepticum* (Supplementary Table 1). The *or*508 gene was designated as *rbsR* based on similarity of the corresponding gene product with the *E. coli* version (Mauzy and Hermodson, 1992). The RbsR protein contains two domains, a N-terminal helix-turn-helix (HTH) DNA binding domain of the LacI family (Pfam PF00356) and a C-terminal ligand-binding domain which is very similar to the sugar-binding domain of an ABC transporter (Pfam PF13377) (Figure 1C). The presence of domains predicted DNA or sugar binding domains therefore suggest that RbsR is a transcriptional factor involved in carbohydrate metabolism (Pérez-Rueda and Collado-Vides, 2000).

Bioinformatic analysis revealed that the genes contiguous with the putative *rbsR* are orthologous with other genes (*rbsD, rbsA, rbsC, rbsB*, and *rbsK*) in the ribose operon (Fineran et al., 2013). The predicted *rbsDACBKR* gene cluster organization in S39006 is the same as in other enterobacteria such as *E. coli, Serratia marcescens* Db11, *D. dadantii, P. atrosepticum, Erwinia amylovora, Yersinia enterocolitica*, and *Klebsiella pneumoniae* (Supplementary Figure 1). In *E. coli*, the *rbsD* gene encodes a ribose mutarotase which converts the β-form of D-ribose into the α-furan form (Ryu et al., 2004); the *rbsABC* genes encode the ABC transporter (in which *rbsA* encodes an ATPase subunit, *rbsB* encodes a periplasmic binding protein and *rbsC* encodes a membrane permease; Park et al., 1999); and the *rbsK* gene encodes the ribokinase which phosphorylates D-ribose to D-ribose-5-phosphate. The *rbsR* gene, encoding the repressor of the *rbsDACBKR* operon, is the terminal gene of the ribose operon (Nentwich et al., 2009).

The phylogenetic relationships of the RbsR proteins in S39006 and 12 taxonomically related strains were analyzed. The phylogenetic tree shows two major clades (Supplementary Figure 2). The RbsR protein in S39006 is monophyletic with the LacI type family transcriptional regulator in *D. dadantii* and closely related to RbsR of *P. atrosepticum*. The RbsR protein is more distantly related to LacI type family transcriptional regulators in other enterobacteria such as *Y. enterocolitica, E. amylovora, K. pneumonia*, and *E. coli*.

### The *rbsR* mutant does not produce GVs

The *rbsR* transposon insertion in S39006 LacA Δ*pigC* was transduced into the wild type S39006 strain to first confirm that the phenotypes observed were due entirely to the transposon. In contrast to wild type S39006 (which is opaque and red) the *rbsR* mutant (transductant) was translucent and non-pigmented on agar plates (Figure 1D). PCM showed no phase-bright structures in the mutant bacterial cells. The phase-bright gas “vacuoles” seen in a wild type strain are aggregates of GVs that appear as bright refractile structures under PCM. GVs were not visible by transmission electron microscopy (TEM) in the *rbsR* mutant (Figure 1E). The absence of GVs was further confirmed using flotation assays. Cultures of wild type S39006 remained buoyant after 48 h, whereas the *rbsR* mutant gradually settled to the bottom of the static liquid culture. Together, these results confirmed that a mutation in *rbsR* repressed GV formation.

The *rbsR* mutation was complemented by a plasmid carrying the wild type allele (pBAD-*rbsR*) and, on induction with arabinose, production of GVs was restored at the indicated arabinose concentrations (Figure 1F). Complemented strains were also restored for flotation and prodigiosin production (Figure 1F, see Supplementary Figure 3D for complementation of additional phenotypes). These results confirmed that the highly pleiotropic phenotypic changes seen in an *rbsR* insertion mutant were fully attributable to the transposon insertion. The *rbsR* gene was also overexpressed in a wild type background but no obvious effects were observed on GV production (data not shown).

**Figure 2:**
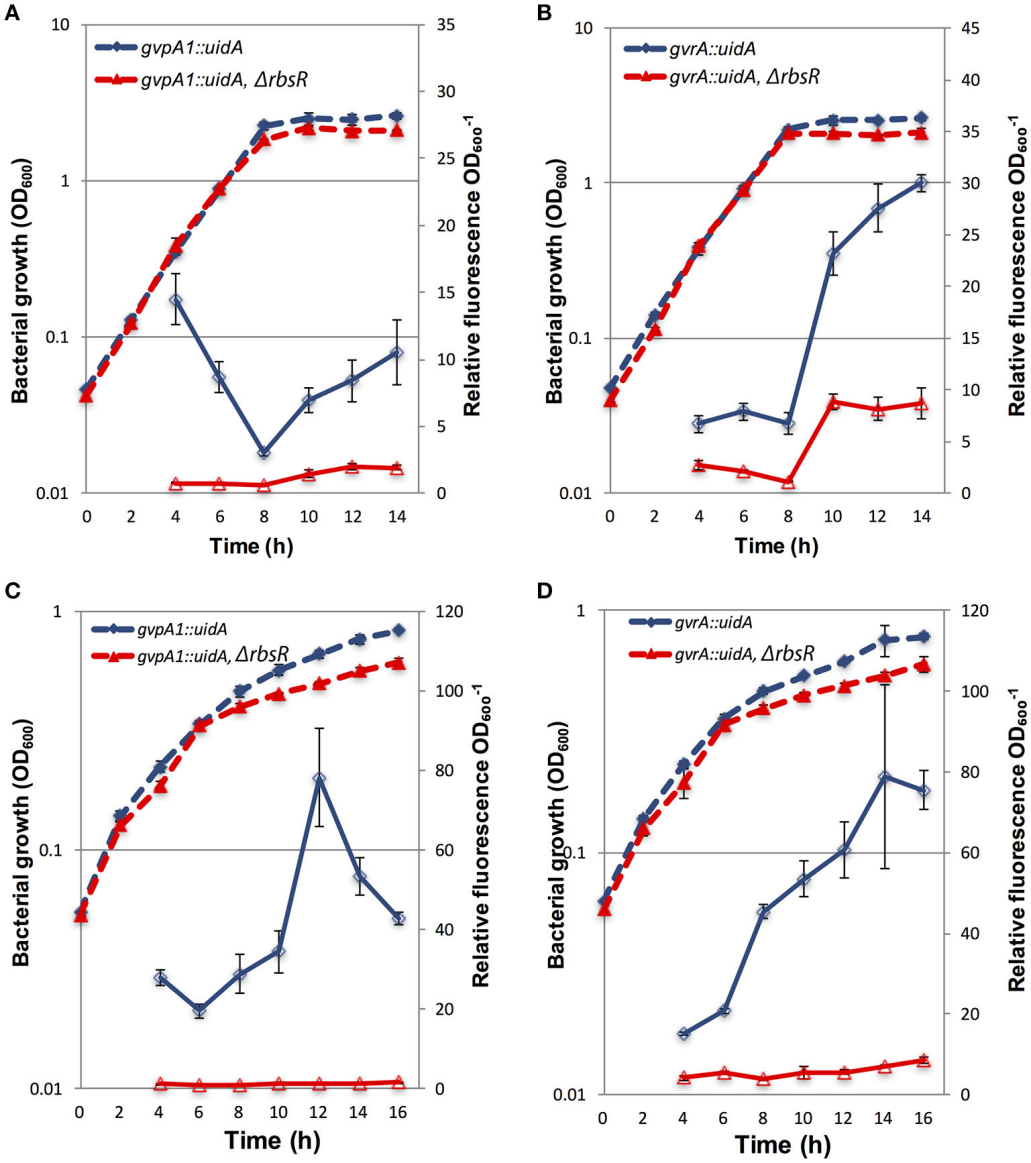
The expression of *gvpA1* and *gvrA* in wild type and *rbsR* backgrounds under aerobic and microaerophilic conditions. β-glu activity from a chromosomal *gvpA1::uidA* fusion strain was assayed in wild type (blue) and the *rbsR* mutant (red) under **(A)** aerobic or **(C)** microaerophilic conditions. β-glu activity was measured from a chromosomal *gvrA::uidA* fusion in the wild type (blue) and *rbsR* background (red) under **(B)** aerobic or **(D)** microaerophilic conditions. Solid lines represent β-glu assays, dashed lines represent the optical density (OD_600_) of wild type and the *rbsR* mutant. Data shown are mean values ± *SD* (*n* = 3). ANOVA two-factor analysis comparing β-glu activity of the indicated fusion in wild type to the *rbsR* mutant throughout growth in **(A)** found *F* = 1344.01 > *F*_*crit*_ = 4.20, *p* = 3.28^*^10^−25^; in **(B)** *F* = 1027.51 > *F*_*crit*_ = 4.20, *p* = 1.29^*^10^−23^; in **(C)** *F* = 990.65 > *F*_*crit*_ = 4.20, *p* = 2.13^*^10^−23^; and in **(D)** *F* = 464.83 > *F*_*crit*_ = 4.20, *p* = 5.61^*^10^−19^.

To investigate regulation by RbsR at the transcriptional level, strains carrying either a chromosomal *gvpA1*::*uidA* or *gvrA*::*uidA* reporter gene fusion were employed. The g*vpA1* and *gvrA* genes are the first genes of the two operons of the GV gene locus (Ramsay et al., 2011; Tashiro et al., 2016). The expression of *gvpA1* or *gvrA* in the wild type and the *rsbR* mutant background was monitored throughout growth using a β-glu assay (a proxy for changes in transcriptional activity from the corresponding promoters). The β-glu activity expressed from *gvpA1*::*uidA* or *gvrA*::*uidA* transcriptional fusions increased between hours eight and 14 in both strains, during stationary phase. However, transcription from the *gvpA1* or *gvrA* promoters in the *rbsR* mutant background was significantly reduced (3–5 fold lower, see ANOVA statistics in Figure Legend) compared to that in S39006 (Figures 2A,B). Similarly, under microaerophilic conditions, transcription of both *gvrA* and *gvpA1* was greatly reduced in an *rbsR* mutant (Figures 2C,D). We also noted a minor growth defect in the *rbsR* mutant when compared to S39006, but this did not affect the data analysis as the β-glu activity was normalized to OD_600_.

### The *rbsK* gene is also negatively regulated by RbsR

The *rbsK* mutant also showed the GV-negative phenotype—as seen in the *rbsR* mutant (Supplementary Figure 3A). This was not unexpected because the transposon, containing a transcriptional terminator (Monson et al., 2015), had been inserted within the *rbsK* gene and so should be polar on the downstream *rbsR* gene. To confirm this predicted polarity, we attempted to complement a mutation in *rbsK* with either one of the plasmids pBAD-*rbsR* or pBAD-*rbsK*. With induction, the plasmid expressing RbsR restored the ability of the *rbsK* mutant to produce GVs and prodigiosin (Supplementary Figure 3A). Further, gene expression from an *rbsK::lacZ* fusion was measured and the expression of *rbsK* was significantly reduced when plasmid-encoded RbsR was induced. (Supplementary Figure 3B). In contrast, the plasmid expressing RbsK was not capable of complementing GV formation or prodigiosin production in a *rbsK* mutant (Supplementary Figure 3C). These results are consistent with RbsR being a negative regulator of the ribose operon in this strain.

### The disruption of *rbsR* has pleiotropic impacts on *Serratia* physiology

**Figure 3:**
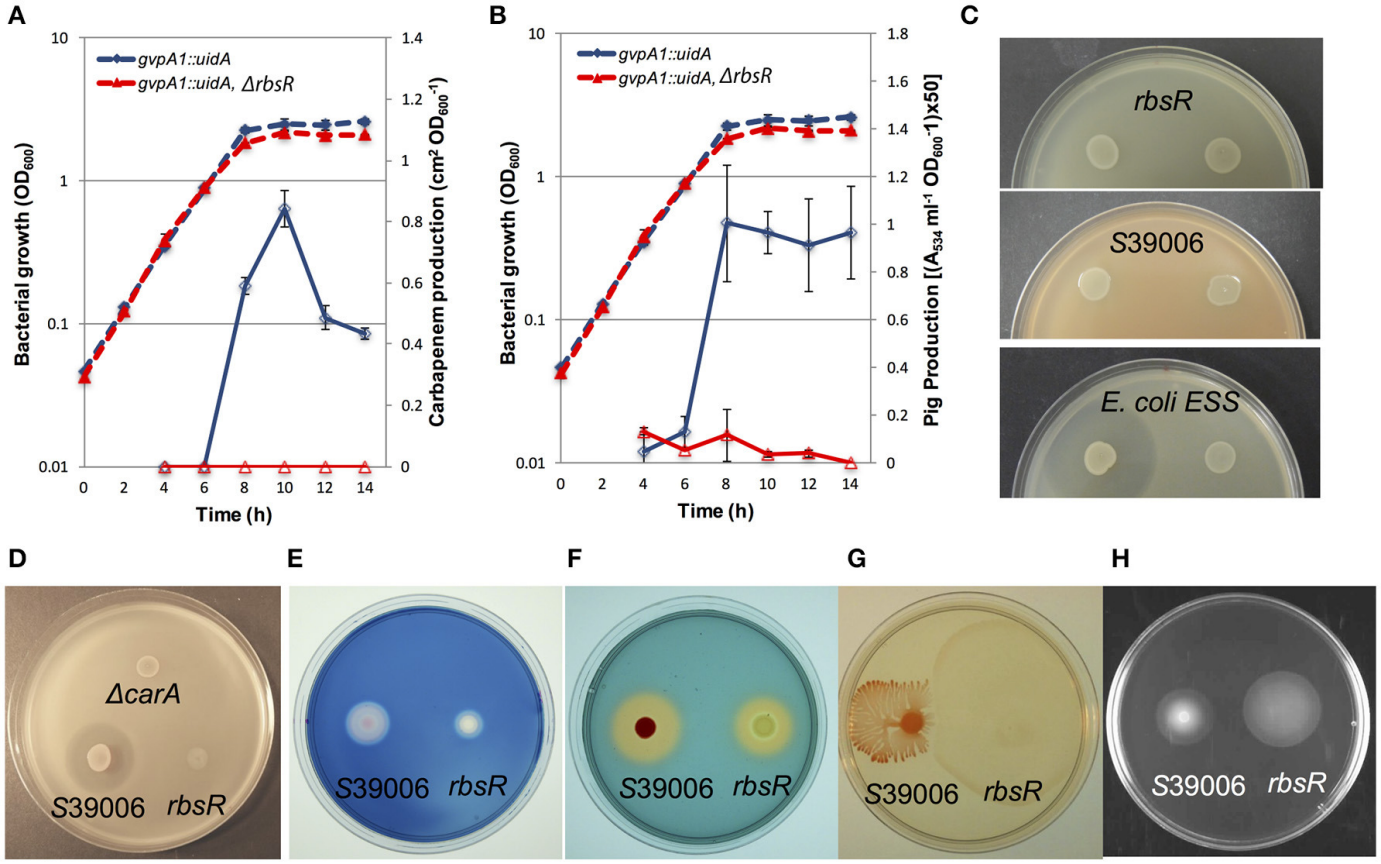
The disruption of *rbsR* has diverse effects on S39006 physiology. The production of **(A)** the carbapenem antibiotic and **(B)** prodigiosin in a *gvpA1::uidA* fusion strain (blue) and the *rbsR* mutant (red) throughout growth in LB. Solid lines represent carbapenem or prodigiosin production and dashed lines represent the optical density (OD_600_) of the bacterial culture. Data shown are means ± *SD* (*n* = 3). **(C)** Carbapenem resistance assay. The carbapenem producing strain *Erwinia carotovora* subsp. *carotovora* (Ecc) ATTN10 (left spot on all plates in **C**) and carbapenem resistant mutant, *Ecc* ATTn10 SM10 (right spot on all plates in **C**) were spotted on the top agar lawn seeded with the *rbsR* mutant (top plate), S39006 (middle plate, positive control), or *E. coli* ESS (bottom plate, negative control) and incubated at 30°C for 48 h. **(D)** Carbapenem production assay of wild type and the *rbsR* mutant; S39006 Δ*carA* was used as a negative control. Test strains with normalized bacterial cell number were spotted on a lawn of *E. coli* ESS and grown at 30°C for 48 h. The production of **(E)** cellulase and **(F)** siderophore, plus **(G)** swarming and **(H)** swimming motility of the wild type and the *rbsR* mutant is shown. Overnight cultures of wild type and the *rbsR* mutant with normalized cell number were spotted on appropriate agars or indicator plates.

S39006 produces bioactive secondary metabolites (a carbapenem and prodigiosin), and PCWDEs, such as cellulase and pectate lyase (Coulthurst et al., 2005; Williamson et al., 2006; Fineran et al., 2007). In addition, S39006 is capable of swimming and swarming (Williamson et al., 2008). Most of these physiological characteristics are under QS control via production of BHL (Thomson et al., 2000; Williamson et al., 2008). A previous study demonstrated that QS also regulated the synthesis of GVs (Ramsay et al., 2011; Ramsay and Salmond, 2012). As the *rbsR* mutation regulated GV and prodigiosin production, we postulated it might also regulate other secondary metabolites and modes of motility. Production of the carbapenem and prodigiosin antibiotics was monitored throughout growth (Figures 3A,B). Both secondary metabolites were impacted: carbapenem antibiotic production was abolished (ANOVA results comparing the wild type and *rbsR*: *F* = 2614.62 > *F*_crit_ = 4.20, *p*-value 3.38^*^10^−29^) and prodigiosin production was significantly reduced in the *rbsR* mutant compared to wild type S39006 (ANOVA results comparing the two strains: *F* = 135.23 > *F*_crit_ = 4.20, *p*-value 3.11^*^10^−12^). Furthermore, in an *rbsR* mutant, activity of a *carA::lacZ* fusion (the first gene in the carbapenem biosynthetic operon) was restored to wild type levels upon expression of RbsR from a plasmid (data not shown).To avoid suicide through carbapenem production, S39006 possesses a carbapenem intrinsic resistance mechanism (Coulthurst et al., 2005). Despite being defective in carbapenem production, the *rbsR* mutant was still resistant to the carbapenem antibiotic as indicated by the absence of a halo of inhibition in resistance assays (Figures 3C,D).

The secretion of cellulase and pectate lyase has been found to contribute to plant pathogenicity of S39006 (Fineran et al., 2007). Similarly, siderophores are produced by many bacteria in an attempt to acquire iron and this can be an important trait for plant virulence (Neilands, 1995). In other plant pathogens, siderophore production has also been found to be under the control of QS (Monson et al., 2013). In the present study, production of both cellulase and the siderophore of S39006 was reduced in the *rsbR* mutant (Figures 3E,F) though no significant changes in production of the QS signaling molecule, BHL, or pectate lyase production were observed (Supplementary Figure 4). Swimming and swarming abilities can also play roles in S39006 virulence (Williamson et al., 2008; Wilf et al., 2013) and the *rbsR* mutant also exhibited increased swimming and swarming motility (Figures 3G,H). We were also able to complement the effects of an *rbsR* mutation on swimming motility, swarming motility, siderophore production and cellulase production by expression of *rbsR* from a plasmid (Supplementary Figure 3D). These observations further highlighted the extent of the pleiotropy caused by the *rbsR* mutation in S39006.

### Identification of the RbsR-binding site by EMSA

**Figure 4:**
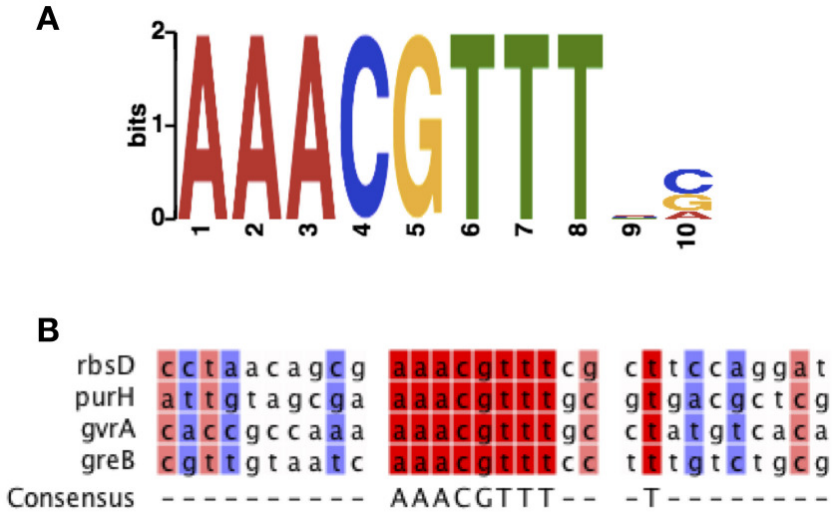
The predicted RbsR-binding motif of S39006. **(A)** Predicted binding sequence of RbsR generated by MEME. **(B)** Sequence alignment of representative binding sequences of RbsR in the S39006 genome.

Bioinformatic analysis revealed that the predicted binding motif of RbsR is conserved among different bacterial lineages (Laikova et al., 2001). The potential DNA binding site of RbsR in S39006 was analyzed using the MEME suite (Bailey et al., 2009). RbsR was predicted to bind to AAACGTTT and this binding motif was found in the upstream region of 18 genes, including *rbsD* and *gvrA*, in S39006 (Figure 4; Supplementary Table 2). The motif was also found upstream of a wide range of genes including those encoding transporters, transcriptional regulators, ATPase, transferase and biosynthetic genes (Supplementary Figure 5; Supplementary Table 2).

**Figure 5:**
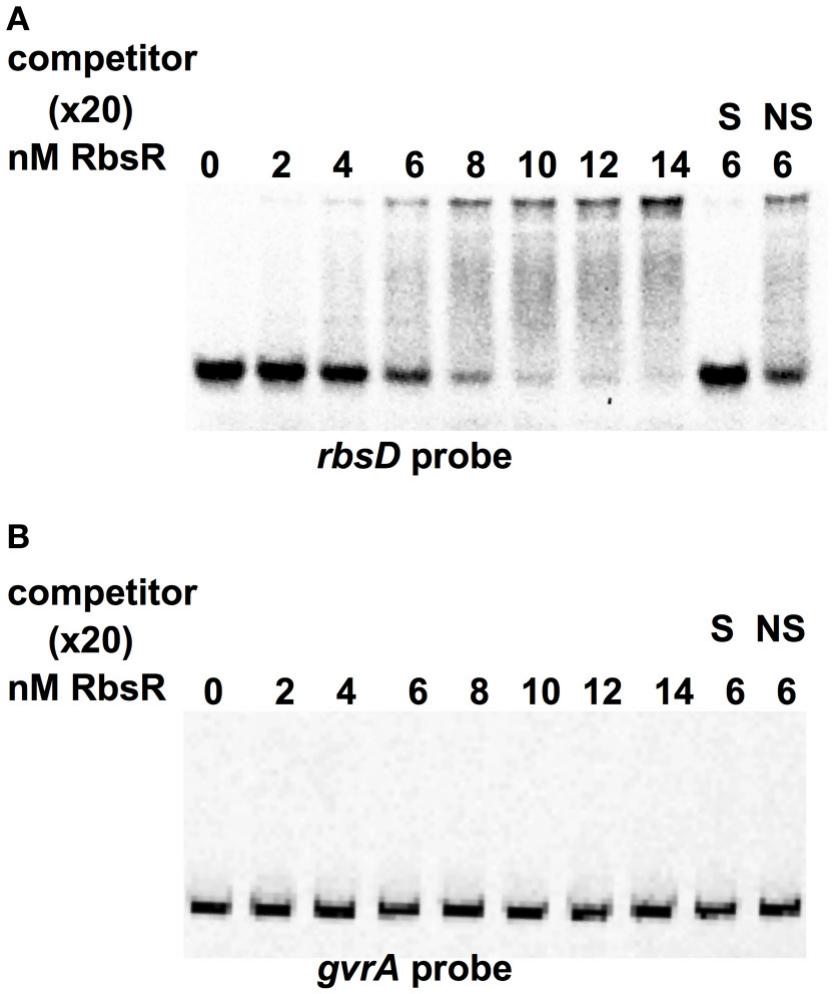
RbsR bound to Cy5-LUEGO-based *rbsD* fragment but not *gvrA* fragment in an EMSA assay. EMSA titrations of the indicated concentration of RbsR protein with **(A)** *rbsD* or **(B)** *gvrA* fragment. The competition experiment was carried out as a control with excess (20X) unlabeled specific (S) probe and nonspecific (NS) probe.

To assay the predicted binding targets of RbsR, EMSA was carried out using the purified RbsR protein. The N-terminal His-tagged RbsR protein was produced. Using the LUEGO method, a gel shift assay with increasing amount of RbsR protein demonstrated that the mobility of the *rbsD* probe was reduced and a complete band-shift was achieved with 14 nM of RbsR (relative ratio protein to DNA of 2.8:1) (Figure 5A). However, there were no discernable changes in the mobility of the *gvrA* probe at the concentrations used (Figure 5B). The EMSA probe was outcompeted by excess (20x) unlabeled probe but not by a non-specific probe, indicating that the binding was specific. Furthermore, in the presence of 1% ribose, RbsR failed to bind to the *rbsD* or *gvrA* probe (Supplementary Figure 5). These results suggest that, under these conditions, ribose is an effector of the RbsR protein.

### The *rbsR* mutant exhibits reduced plant virulence

**Figure 6:**
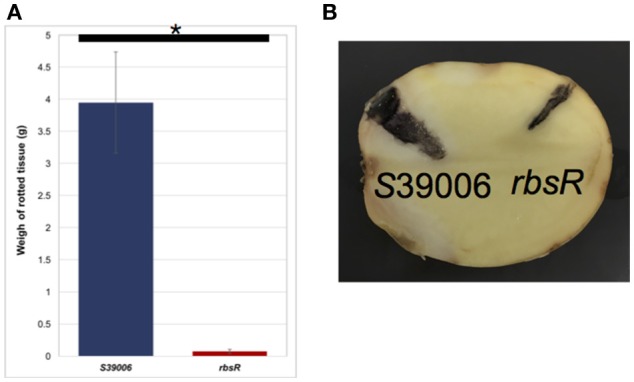
The *rbsR* mutation reduced plant virulence compared to wild type. **(A)** Comparison of rotted tissues produced by wild type and the *rbsR* mutant after 5 days of incubation with initial inoculation of 10^6^ cfu of bacterial cells. Values are the average of three biological replicates and error bars indicate ± *SD*, asterisk indicates significant differences (*t*-test, *p* < 0.05). **(B)** Representative potato with rotted tissue areas (stained by iodine to enhance clarity) injected with either wild type or the *rbsR* mutant.

To assess any impacts on plant virulence, potato rotting assays of S39006 and the *rbsR* mutant were performed. Potato tubers were inoculated with 1 × 10^6^ cfu of bacterial cells and incubated at 30°C for 5 days. The rotted tissue produced by the *rbsR* mutant was significantly lower than that produced by the wild type (Figure 6). This observation is consistent with the cellulase plate assay which showed significant reduction in elaboration of this virulence determinant (Figure 3C). However, the bacterial viable cell count revealed that the mutant exhibited impaired growth in potato tubers. For wild type cells, the number of colony forming units per tenth of a gram of rot was always greater than 10^8^. In contrast, for the *rbsR* mutant, colony numbers never exceeded 10^4^ for the same weight of inoculated potato rot.

## Discussion

S39006 is the first and, thus far, only enterobacterium shown to produce functional GVs naturally (Ramsay et al., 2011; Tashiro et al., 2016). The S39006 GV gene locus comprises 19 genes which are subdivided into two operons (Ramsay et al., 2011; Tashiro et al., 2016). A previous study has shown that 11 genes are essential to produce robust GVs. Functional production of GVs also requires maintenance of each GV gene product at correct stoichiometric levels (Monson et al., 2016). Previous studies have shown that the production of GVs in S39006 involves a multifactorial regulatory network. QS and the Rsm system co-regulate GVs, secondary metabolites and exoenzyme production, as well as motility in S39006 (Thomson et al., 2000; Coulthurst et al., 2005; Fineran et al., 2007; Williamson et al., 2008; Ramsay et al., 2011). However, there is still a comparative paucity of information on factors regulating GV biogenesis in S39006. In this study we have identified a novel regulator, RbsR, which plays an important role in GV morphogenesis. Further, we have shown that, in addition to GV production, the *rbsR* gene also regulates carbapenem, prodigiosin, siderophore, and cellulase production as well as swarming and swimming motility. In previous studies, the complex hierarchical regulatory networks of carbapenem and prodigiosin biosynthesis have been studied intensively (Coulthurst et al., 2005; Williamson et al., 2006). Consequently, it is perhaps surprising that the *rbsR* gene was not identified previously in screens for regulators of either antibiotic. The present study also defines a new link between a carbon source, ribose, and the control of carbapenem and prodigiosin antibiotic production.

The *rbsR* gene in S39006 is predicted to encode the repressor of the ribose operon. The *rbsR* gene is located downstream of *rbsK* and is the last gene of the *rbsDACBKR* operon, responsible for the high affinity transport of D-ribose (Mauzy and Hermodson, 1992). The *rbsR* gene in *Serratia* spp. has not been studied previously but it has been well-characterized in *E. coli* (Mauzy and Hermodson, 1992; Shimada et al., 2013). The ribose operon configuration of S39006 is the same as in *E. coli* (Supplementary Figure 1) implying similar gene regulation. The RbsR protein in *E. coli* is a negative regulator of the ribose operon (Lopilato et al., 1984; Mauzy and Hermodson, 1992). In S39006, providing the *rbsR* gene *in trans* reduced the expression of the *rbsK* gene significantly, indicating negative control by RbsR (Supplementary Figure 3) confirming the predicted function of RbsR as the repressor of the ribose operon.

The RbsR protein belongs to the LacI family and bioinformatic analysis suggests that the binding motifs of this protein are highly conserved among bacteria (Milk et al., 2010). This family of regulators primarily acts as repressors in carbon metabolism. However, in a bioinformatics study, about 20% of the 1303 LacI family transcription factors analyzed were predicted to bind to more than one binding motif (Ravcheev et al., 2014). In the present study, similar binding sites of RbsR (AAACGTTT) were predicted to lie 5′ of 18 genes in S39006, including *rbsD* and *gvrA*. This is in agreement with the binding motifs of the LacI family transcriptional factors which are palindromes and contain a conserved inverted repeat (Camas et al., 2010). RbsR bound to the upstream region of *rbsD* in S39006, as predicted. Similarly, in *E. coli*, the RbsR protein also bound to DNA sequences upstream of *rbsD* (Ryu et al., 2004). In a more recent report, the RbsR repressor was also shown to bind to predicted promoter regions of *add* and *udk* to regulate the salvage pathway of nucleotide synthesis. In addition, RbsR also negatively regulates the *purHD* operon for purine nucleotide metabolism (Shimada et al., 2013). Most LacI family transcription factors are regulators of linked carbohydrate metabolism genes, however, some (such as CcpA, FruR, and PurR) regulate a diverse set of metabolic pathways in bacteria (Ravcheev et al., 2014). In S39006, we have shown that RbsR bound upstream of *rbsD* and the predicted RbsR binding motif was identified upstream of several genes in the genome. Therefore, RbsR could potentially act as a global regulator controlling multiple metabolic pathways in S39006.

Currently, most reports on RbsR have focused on ribose transport and nucleotide metabolism. In this study, however, we showed that a mutation in the *rbsR* gene had a pronounced impact on the biosynthesis of GVs and the *rbsR* mutation significantly reduced the transcription of *gvpA1* and *gvrA*. Because a predicted RbsR binding motif was found upstream of *gvrA* the RbsR protein was predicted to bind to this region and activate production of GVs. However, despite strong conservation of the predicted binding site and despite multiple attempts under various conditions, EMSA failed to show any binding of RbsR upstream of *gvrA*. One possible reason could be that binding requires additional transcriptional (co)-factors at this target site. To regulate gene transcription, many transcriptional factors need cofactors or other transcriptional factors to work cooperatively. For example, in *Bacillus, Clostridium*, and *Lactobacillus*, CRE boxes were predicted close to the RbsR binding sites and this indicated the potential involvement of catabolite control protein A (CcpA) (Rodionov et al., 2001). However, the binding of CcpA to the CRE boxes has not been proven experimentally. A mutation in *ccpA* in *Bacillus subtilis* was found to relieve catabolite repression of the *rbs* operon in a carbon-limiting environment (Strauch, 1995). We are forced to conclude that either RbsR does not bind at all to the *gvrA* upstream sequence (which seems unlikely given the strong conservation of the putative RbsR binding sequence predicted in this region) or the factors affecting binding of RbsR at the *gvrA* site may be more biochemically complex than required for binding at the “control” site—upstream of the ribose operon.

In S39006, ribose is an effector of RbsR as indicated by the release of binding upon the addition of ribose in the EMSA assays. In *E. coli*, ribose is also the negative effector of the homologous RbsR (Mauzy and Hermodson, 1992). Similarly, ribose was also found to de-repress the *rbsUDK* operon in *Staphylococcus aureus*, by Northern blotting analysis (Lei and Lee, 2015). When sugar binds to the C-terminal ligand-binding domain, the repressor undergoes a conformational change which causes a reduction in the DNA binding affinity (Fukami-Kobayashi et al., 2003). Similarly, we found that addition of ribose to the purified RbsR protein of S39006 altered the DNA binding affinity of the protein, suggesting that the sugar does indeed affect protein conformation.

In aquatic environments, S39006 is predicted to have an adaptive advantage by allowing GV-mediated flotation into more oxygenated niches. The expression of GV genes in S39006 is modulated by oxygen concentration; high expression of *gvpA1* was consistently observed in S39006 cultures with limited oxygenation (Ramsay et al., 2011). In this study, we investigated whether a mutation in *rbsR* affected GV gene expression in an oxygen-dependent manner. Transcription of a *gvpA1* fusion in the *rbsR* mutant was reduced under microaerophilic conditions implying that *rbsR*–mediated regulation of GVs is independent of oxygen tension.

A mutation in the *rbsR* gene not only affected GV biosynthesis but also caused diverse impacts on S39006 physiology. One possible explanation for these observations was that RbsR regulated QS. QS is known to regulate GV production through direct transcriptional repression of *gvpA1* (Tashiro et al., 2016) though it is also known to control many other phenotypes in S39006 (Thomson et al., 2000; Williamson et al., 2008). However, in the biosensor assay for BHL production, the *rbsR* mutant and wild type S39006 produced similar levels of the QS signaling molecule. Consequently, there is no evidence for involvement of QS in the RbsR-dependent regulation of GV production and other phenotypes.

We also noted that, while carbapenem production was abolished in an *rbsR* mutant, it remained resistant to the antibiotic. Biosynthesis of the carbapenem is encoded by the *carA-H* operon. The first five genes of the operon (*carA, B, C, D*, and *E*) are responsible for biosynthesis of the carbapenem; *carF* and *G* encode components involved in an intrinsic β-lactam resistance mechanism (to avoid suicide in the producer host) and the function of *carH* is unknown (Coulthurst et al., 2005; Tichy et al., 2014). The carbapenem operon is under the control of CarR, a LuxR-type regulator but the intrinsic resistance genes are also driven independently by an internal promoter to ensure that they are expressed constitutively. In S39006, the *rbsR* mutation has an impact on the biosynthesis of, but not intrinsic resistance to, the carbapenem—presumably by indirect impacts via the main promoter of the carbapenem operon but with no down-regulation of the intrinsic resistance genes driven by the internal promoter in the *carA*-*H* operon (Coulthurst et al., 2005).

In a plant virulence assay, the *rbsR* mutation diminished soft rot of potato tissue. The mutant produced significantly less cellulase and this may have impacted on growth in potato tubers. Changes in motility are also likely to have impacted on plant virulence. In other plant pathogens motility is a requirement for virulence (Mulholland et al., 1993; Tans-Kersten et al., 2001; Haiko and Westerlund-Wikstrom, 2013). However, in an *rbsR* mutant, motility was increased but potato tuber virulence was decreased. This suggests that the increase in motility could not overcome the growth defect in this strain or that the conditions used to assess cellular motility (such as swarming or swimming plate assays) may not be representative of motility in potato tissue. Interestingly, in a recent report, RbsR was shown to activate capsule genes (encoding an important virulence factor) in the human pathogen, *Staphylococcus*, where RbsR bound to the *cap* promoter and activated *cap* gene expression (Lei and Lee, 2015). By analogy therefore, RbsR is potentially a “global” regulator affecting plant virulence, either acting through the existing regulators of virulence in S39006 such as Rap, PigX, or the PstSCAB-PhoBR system or through new, as yet unidentified, regulators (Williamson et al., 2006; Fineran et al., 2007; Gristwood et al., 2009).

In summary, we have shown that RbsR is a novel regulator for GV biosynthesis in S39006. The results of the study also suggest that a new carbon source—ribose—is potentially involved in controlling prodigiosin and carbapenem antibiotic production. In addition, this study has uncovered diverse physiological effects of the *rbsR* mutation in siderophore and cellulase production as well as swarming and swimming motility. This strongly pleiotropic role for RbsR (even in a series of phenotypes that are already well-characterized) strongly implies that the metabolic impacts of this regulator will be even more dramatic than are currently known. Consequently, future work will focus on investigating the physiological panorama of RbsR-dependent metabolism in S39006 using a variety of ‘omics approaches.

## Author contributions

CL, RM, and RA performed experiments. CL and RM analyzed data and wrote the manuscript. CL, RA, RM, and GS designed experiments. RM and GS edited the manuscript.

### Conflict of interest statement

The authors declare that the research was conducted in the absence of any commercial or financial relationships that could be construed as a potential conflict of interest. The reviewer GJ and handling Editor declared their shared affiliation, and the handling Editor states that the process nevertheless met the standards of a fair and objective review.

## Supplementary material

Supplementary Figure 1 Comparison of the ribose operon genetic organization in S39006 with selected closely related strains. ORFs of similar predicted functions are indicated by arrow blocks in the same color. The scale bar represents 1 Kb.

Supplementary Figure 2 Molecular phylogenetic analysis of the RbsR protein by the Maximum Likelihood method. The evolutionary history was inferred by using the Maximum Likelihood method based on the JTT matrix-based model. Evolutionary analyses were conducted in MEGA7 (Kumar et al., 2016).

Supplementary Figure 3 The *rbsK* mutant, like *rbsR* mutant, does not produce GVs. **(A)** Complementation of the *rbsK* mutation with a plasmid encoding an *rbsR* gene in increasing concentrations of arabinose. The top image shows flotation assays of wild type, the *rbsK* mutant carrying the empty vector pBAD30, or the *rbsK* mutant carrying pBAD-*rbsR*. The middle image is the bacterial patches on an agar plate. The bottom image shows PCM images of bacterial cells from agar plates. **(B)** β-glu activity from a chromosomal *rbsK::lacZ* fusion strain with or without the plasmid pBAD-*rbsR* at 0 and 0.2% arabinose. **(C)** RbsK cannot complement gas vesicle formation in an *rbsK* mutant. Control PCM images of LacA containing pBAD33 are shown in the top row. An *rbsK* mutant with either pBAD33 (middle row) or pBAD-*rbsK* (bottom row) grown in the indicated arabinose concentration are shown imaged by PCM and on agar plates by colony opacity. In each case, these are representative images. The scale bar at the bottom of each PCM image indicates 1 μm **(D)** Complementation of swimming motility (top row), swarming motility (second row), siderophore production (third row), and cellulase activity (bottom row) by a plasmid expressed copy of RbsR. In each plate, both strains are carry the same mutation in rbsR, but the colony on the left contains the vector pBAD30 and on the right pBAD-*rbsR*. A normalized number of cells were grown on the indicated assay plates with either no additional arabinose (left column) or 0.2% arabinose (right column).

Supplementary Figure 4 Phenotypic characterization of the wild type and the *rbsR* mutant. **(A)** Wild type and the *rbsR* mutant produce similar level of BHL (quorum sensing molecule). Ten microliters of normalized bacterial cell number of wild type and the *rbsR* mutant was spotted on a lawn of *Serratia* biosensor strain SP19 and *Serratia* LIS (BHL defective strain) was used as negative control. **(B)** Pectate lyase production of the wild type and the *rbsR* mutant. Test strains with normalized bacterial cell number were spotted on appropriate indicator plate.

Supplementary Figure 5 Gel shift experiments of RbsR with **(A)** *rbsD* or **(B)** *gvrA* probe in the presence of 1% ribose. The competition experiment was carried out as control with excess (20X) unlabeled specific (S) probe and nonspecific (NS) probe.

Supplementary Table 1 Amino acids sequence similarity search of RbsR using BLAST by the Genbank.

Supplementary Table 2 The locations of predicted binding of the RbsR repressor.
