# Supplementary figures and images for: The LacI–Family Transcription Factor, RbsR, Is a Pleiotropic Regulator of Motility, Virulence, Siderophore and Antibiotic Production, Gas Vesicle Morphogenesis and Flotation in Serratia
Source: Front Microbiol. 2017 Sep 11;8:1678. doi: 10.3389/fmicb.2017.01678 (PMC5601083; doi:10.3389/fmicb.2017.01678)

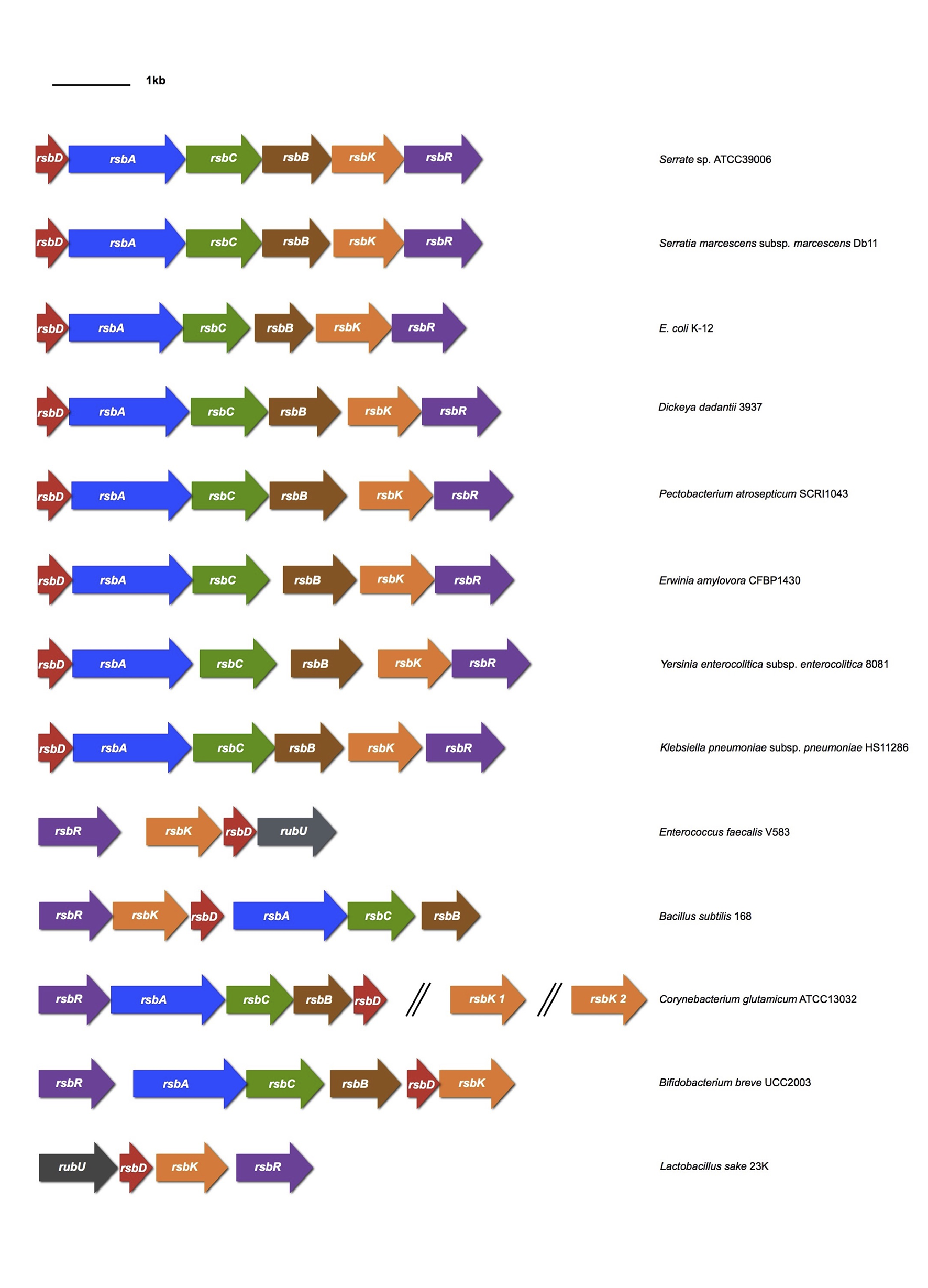

Supplement: Supplementary Figure 1 — Comparison of the ribose operon genetic organization in S39006 with selected closely related strains. ORFs of similar predicted functions are indicated by arrow blocks in the same color. The scale bar represents 1 Kb. [file Image1.TIFF]

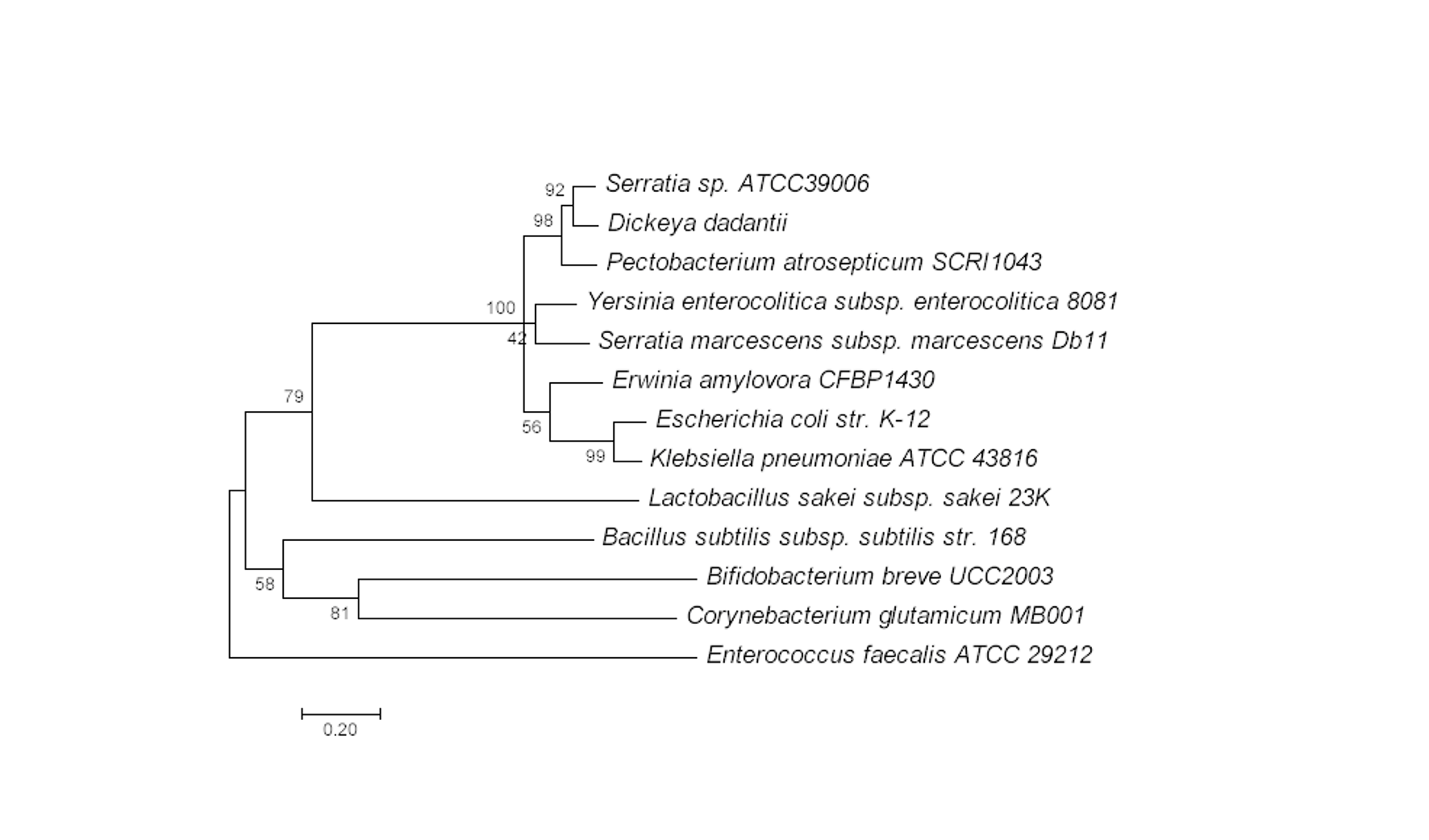

Supplement: Supplementary Figure 2 — Molecular phylogenetic analysis of the RbsR protein by the Maximum Likelihood method. The evolutionary history was inferred by using the Maximum Likelihood method based on the JTT matrix-based model. Evolutionary analyses were conducted in MEGA7 (Kumar et al., 2016). [file Image2.TIFF]

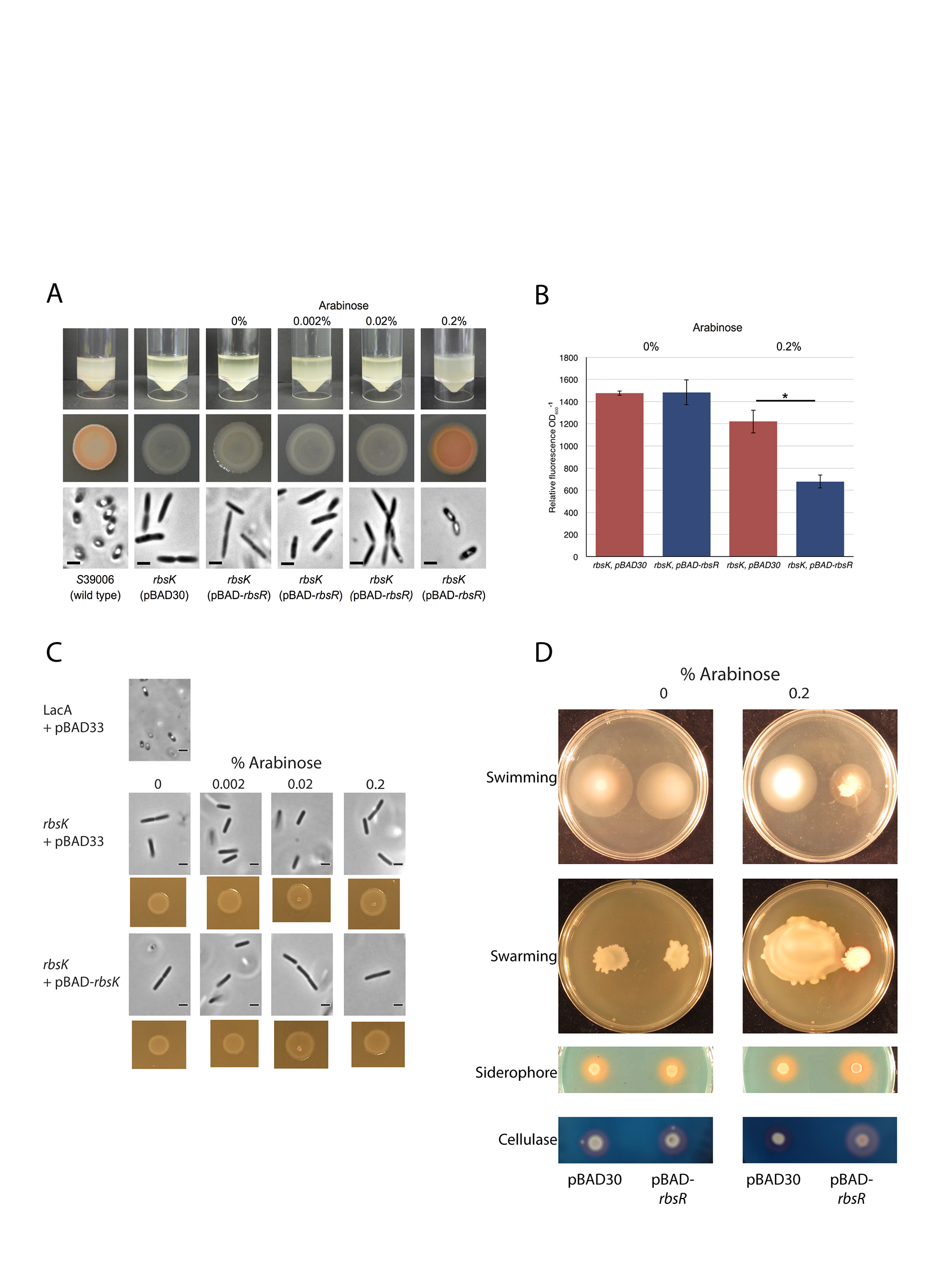

Supplement: Supplementary Figure 3 — The rbsK mutant, like rbsR mutant, does not produce GVs. (A) Complementation of the rbsK mutation with a plasmid encoding an rbsR gene in increasing concentrations of arabinose. The top image shows flotation assays of wild type, the rbsK mutant carrying the empty vector pBAD30, or the rbsK mutant carrying pBAD-rbsR. The middle image is the bacterial patches on an agar plate. The bottom image shows PCM images of bacterial cells from agar plates. (B) β-glu activity from a chromosomal rbsK::lacZ fusion strain with or without the plasmid pBAD-rbsR at 0 and 0.2% arabinose. (C) RbsK cannot complement gas vesicle formation in an rbsK mutant. Control PCM images of LacA containing pBAD33 are shown in the top row. An rbsK mutant with either pBAD33 (middle row) or pBAD-rbsK (bottom row) grown in the indicated arabinose concentration are shown imaged by PCM and on agar plates by colony opacity. In each case, these are representative images. The scale bar at the bottom of each PCM image indicates 1 μm (D) Complementation of swimming motility (top row), swarming motility (second row), siderophore production (third row), and cellulase activity (bottom row) by a plasmid expressed copy of RbsR. In each plate, both strains are carry the same mutation in rbsR, but the colony on the left contains the vector pBAD30 and on the right pBAD-rbsR. A normalized number of cells were grown on the indicated assay plates with either no additional arabinose (left column) or 0.2% arabinose (right column). [file Image3.TIF]

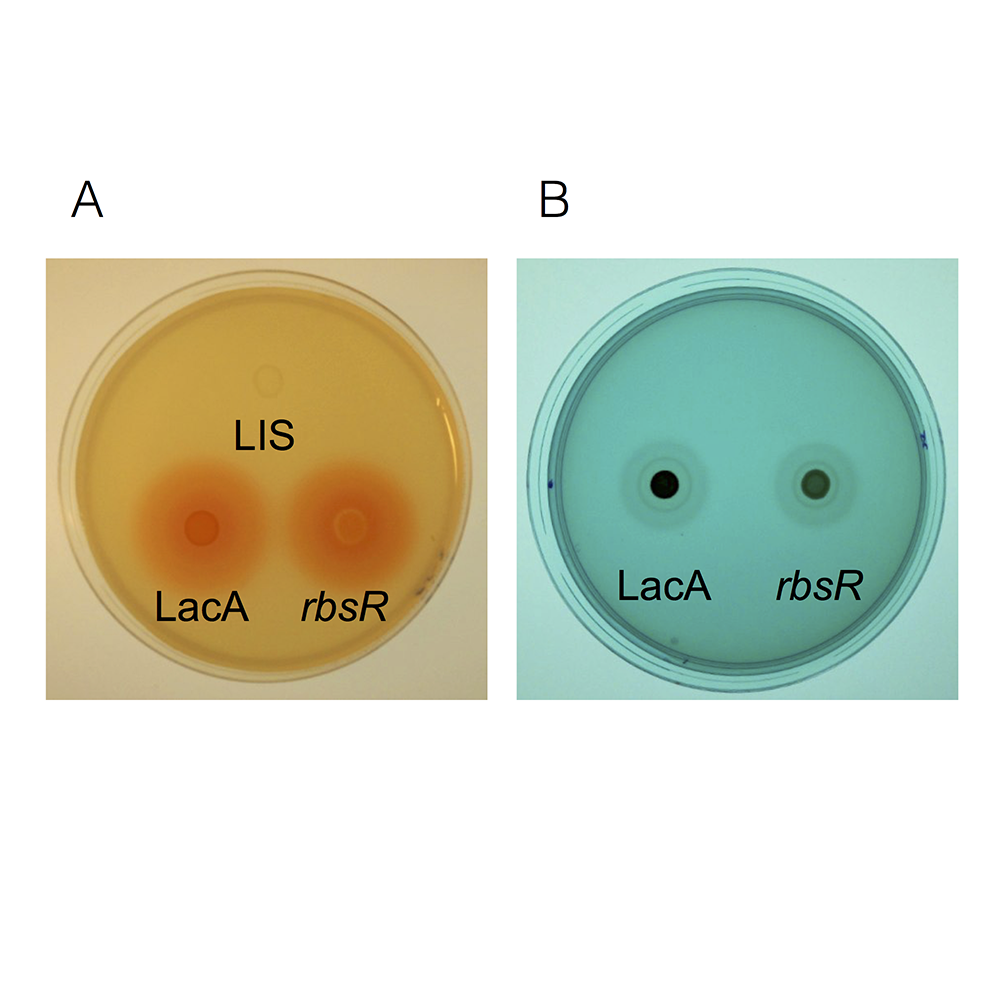

Supplement: Supplementary Figure 4 — Phenotypic characterization of the wild type and the rbsR mutant. (A) Wild type and the rbsR mutant produce similar level of BHL (quorum sensing molecule). Ten microliters of normalized bacterial cell number of wild type and the rbsR mutant was spotted on a lawn of Serratia biosensor strain SP19 and Serratia LIS (BHL defective strain) was used as negative control. (B) Pectate lyase production of the wild type and the rbsR mutant. Test strains with normalized bacterial cell number were spotted on appropriate indicator plate. [file Image4.TIFF]

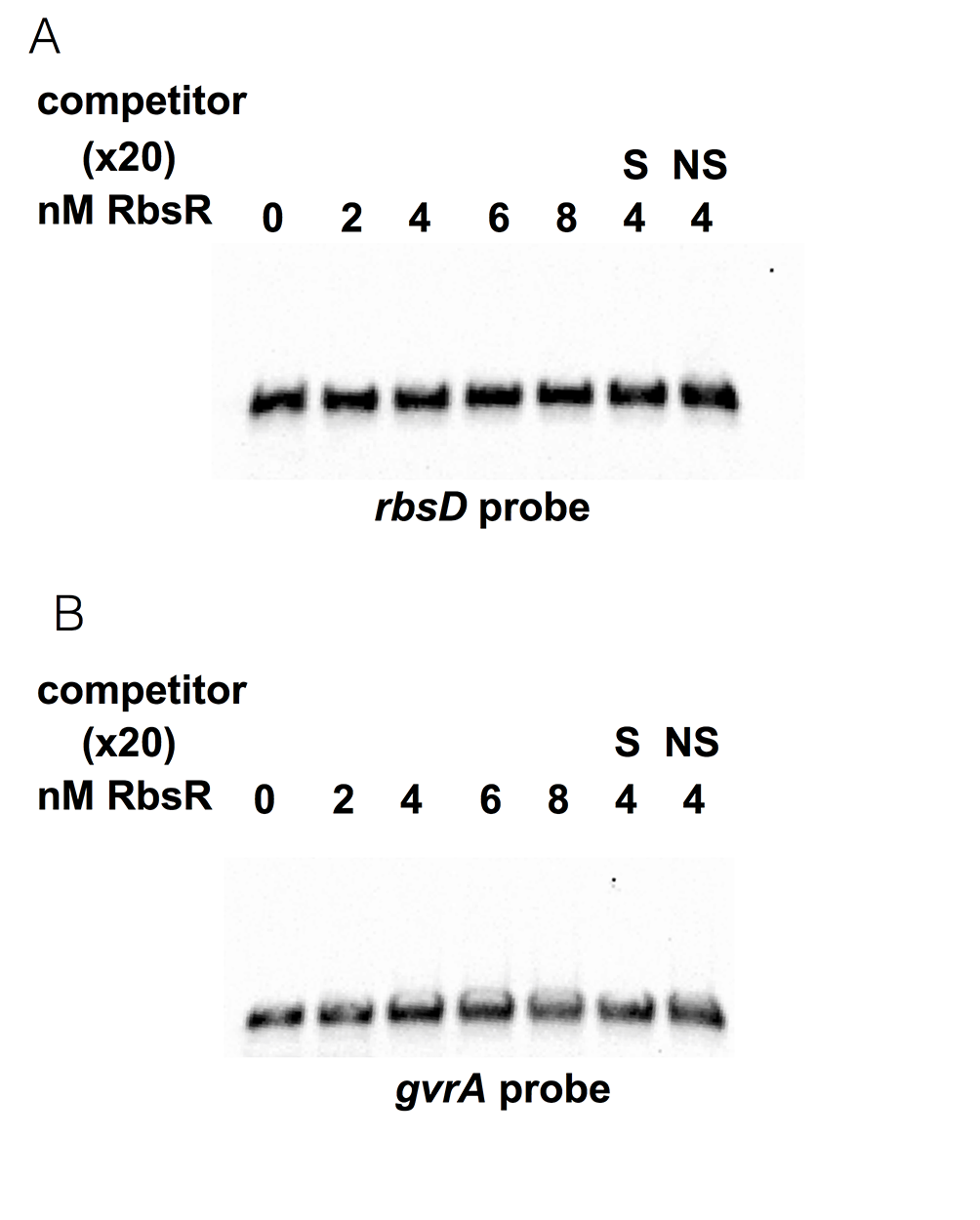

Supplement: Supplementary Figure 5 — Gel shift experiments of RbsR with (A) rbsD or (B) gvrA probe in the presence of 1% ribose. The competition experiment was carried out as control with excess (20X) unlabeled specific (S) probe and nonspecific (NS) probe. [file Image5.TIFF]
