# Supplementary material for: The LacI–Family Transcription Factor, RbsR, Is a Pleiotropic Regulator of Motility, Virulence, Siderophore and Antibiotic Production, Gas Vesicle Morphogenesis and Flotation in Serratia
Source: Front Microbiol. 2017 Sep 11;8:1678. doi: 10.3389/fmicb.2017.01678 (PMC5601083; doi:10.3389/fmicb.2017.01678)
Supplement: Supplementary Table 1 — Amino acids sequence similarity search of RbsR using BLAST by the Genbank. [file Table1.DOCX]

**Supplementary Table 1.** Amino acids sequence similarity search of RbsR using BLAST by the Genbank.

| Species | Identity (%) | Similarity(%) |
| --- | --- | --- |
| *Dickeya dadantii* | 89 | 95 |
| *D. dianthicola* | 88 | 94 |
| *D. solani* | 88 | 93 |
| *D. zeae* | 88 | 94 |
| *D. chrysanthemi* | 87 | 93 |
| *Pectobacterium atrosepticum* | 86 | 93 |
