# Supplementary material for: The LacI–Family Transcription Factor, RbsR, Is a Pleiotropic Regulator of Motility, Virulence, Siderophore and Antibiotic Production, Gas Vesicle Morphogenesis and Flotation in Serratia
Source: Front Microbiol. 2017 Sep 11;8:1678. doi: 10.3389/fmicb.2017.01678 (PMC5601083; doi:10.3389/fmicb.2017.01678)
Supplement: Supplementary Table 2 — The locations of predicted binding of the RbsR repressor. [file Table2.DOCX]

| No. | Name | Predicted function | *p*-value | Sites |
| --- | --- | --- | --- | --- |
| 1 | *greB* | transcription elongationfactor | 1.17e-6 | CGTTGTAATCAAACGTTTCCTTTGTCTGCG |
| 2 | or0026 | oxoglutarate/malate translocator | 1.17e-6 | GCGTATTAGCAAACGTTTCCGACCTCAGCA |
| 3 | *nhaA* | pH dependent sodium/proton antiporter | 4.11e-6 | GAATGCCGGAAAACGTTTACGAAGAGATAA |
| 4 | *wza* | capsule polysaccharide export protein | 4.11e-6 | CCAATTTATAAAACGTTTACAATAAACATT |
| 5 | or3402 | colicin V production protein | 4.11e-6 | CAGAAAAAGAAAACGTTTGCGTAGCGGATT |
| 6 | *deoC* | deoxyribose phosphate aldolase | 4.11e-6 | GGATATCACCAAACGTTTACGTTACCCGTG |
| 7 | *purH* | bifunctional purine biosynthesis protein | 4.11e-6 | ATTGTAGCGAAAACGTTTGCGTGACGCTCG |
| 8 | *gvrA* | response regulator receiver protein | 4.11e-6 | CACCGCCAAAAAACGTTTGCCTATGTCACA |
| 9 | or2059 | glycoside hydrolase clan GH-D | 5.28e-6 | TCTTTACCCCAAACGTTTCGGAATAAAACC |
| 10 | *rbsD* | D-ribose pyranase | 5.28e-6 | CCTAACAGCGAAACGTTTCGCTTCCAGGAT |
| 11 | or2686 | extracellular solute-binding protein family 5 | 8.22e-6 | CTCTGATGTCAAACGTTTGGTGCTTTAAGC |
| 12 | or1014 | transport-associated protein | 8.22e-6 | TATGAGAAAAAAACGTTTAGAGAACGATTT |
| 13 | or0032 | homoserine o-succunyltransferase | 8.22e-6 | GCATACGTATAAACGTTTAGACATCCAGAT |
| 14 | *ada* | bifunctional transcriptional regulator of DNA repair | 1.00e-5 | CGGCACGGTAAAACGTTTTCAATAATAAGT |
| 15 | or01924 | winged helix family two component transcriptional regulator | 1.18e-5 | TTAAGTCAAGAAACGTTTTGTGGTTCTT |
| 16 | or2255 | sugar efflux transporter | 1.36e-5 | AAAACCGCTGAAACGTTTCAAAAATTCTGA |
| 17 | *zntA* | zinc/cadmium/mercury/lead-transporting ATPase | 1.80e-5 | AAAACGTTTAAAACGTTTAAAAATAGAGCG |
| 18 | or0493 | FOF1 ATP synthase subunit C | 2.08e-5 | TCAACACTACAAACGTTTACATTGAAAAAA |

**Supplementary Table 2.** The locations of predicted binding of the RbsR repressor.
